# Increased brain expression of *GPNMB* is associated with genome wide significant risk for Parkinson’s disease on chromosome 7p15.3

**DOI:** 10.1007/s10048-017-0514-8

**Published:** 2017-04-08

**Authors:** Megha N. Murthy, Cornelis Blauwendraat, UKBEC, Sebastian Guelfi, IPDGC, John Hardy, Patrick A. Lewis, Daniah Trabzuni

**Affiliations:** 1 Genetics and Genomics Laboratory, DOS in Genetics and Genomics, University of Mysore, Mysore, Karnataka 570006 India; 2 School of Pharmacy, University of Reading, Whiteknights, Reading, RG6 6AP UK; 3 Neurodegenerative Diseases Research Unit, National Institute of Neurological Disorders and Stroke, National Institutes of Health, Bethesda, MD USA; 4 Department of Molecular Neuroscience, UCL Institute of Neurology, Queen Square, London, WC1N 3BG UK; 5 Department of Genetics, King Faisal Specialist Hospital and Research Centre, Riyadh, 11211 Saudi Arabia

**Keywords:** Chr7 locus (*GPNMB*), Antisense and non-coding RNA, Human brain expression QTLs, Parkinson’s disease (PD), Risk SNP rs199347

## Abstract

Genome wide association studies (GWAS) for Parkinson’s disease (PD) have previously revealed a significant association with a locus on chromosome 7p15.3, initially designated as the glycoprotein non-metastatic melanoma protein B (*GPNMB*) locus. In this study, the functional consequences of this association on expression were explored in depth by integrating different expression quantitative trait locus (eQTL) datasets (Braineac, CAGEseq, GTEx, and Phenotype-Genotype Integrator (PheGenI)). Top risk SNP rs199347 eQTLs demonstrated increased expressions of *GPNMB*, *KLHL7*, and *NUPL2* with the major allele (AA) in brain, with most significant eQTLs in cortical regions, followed by putamen. In addition, decreased expression of the antisense RNA *KLHL7-AS1* was observed in GTEx. Furthermore, rs199347 is an eQTL with long non-coding RNA (*AC005082.12*) in human tissues other than brain. Interestingly, transcript-specific eQTLs in immune-related tissues (spleen and lymphoblastoid cells) for *NUPL2* and *KLHL7-AS1* were observed, which suggests a complex functional role of this eQTL in specific tissues, cell types at specific time points. Significantly increased expression of *GPNMB* linked to rs199347 was consistent across all datasets, and taken in combination with the risk SNP being located within the *GPNMB* gene, these results suggest that increased expression of *GPNMB* is the causative link explaining the association of this locus with PD. However, other transcript eQTLs and subsequent functional roles cannot be excluded. This highlights the importance of further investigations to understand the functional interactions between the coding genes, antisense, and non-coding RNA species considering the tissue and cell-type specificity to understand the underlying biological mechanisms in PD.

**Electronic supplementary material:**

The online version of this article (doi:10.1007/s10048-017-0514-8) contains supplementary material, which is available to authorized users.

## Introduction

Parkinson’s disease (PD) is the second most common neurodegenerative disease, characterized by movement-related symptoms including bradykinesia, rigidity, and tremor, as well as an increasingly appreciated array of non-movement issues [1]. The symptoms derive from extensive neuronal cell death, most notably (but not exclusively) of dopaminergic neurons within the *substantia nigra pars compacta*. The etiology of PD is complex, and is thought to involve the interplay of several factors, including environmental exposure and genetic predisposition. Our understanding of the latter has undergone a transformation in the last two decades, moving from fully penetrant causative variants inherited in a Mendelian fashion to subtle risk factors impacting on transcript expression, gene-gene interactions, gene-protein interactions, and other downstream processes in different tissues and specific cell type [2]. In recent years, genome wide association (GWA) meta-analyses have opened a new window on how common variation in the general population can increase lifetime risk of developing PD. The most recent of these, a meta-analysis study conducted by Nalls et al. included 13,708 cases and 95,282 controls, identified 26 risk loci of which 6 were novel. Thirty significant associations between SNPs of interest and either CpG methylation or messenger RNA (mRNA) expression profiles across the six newly identified loci were identified [3]. Thus, the application of GWA approaches over the past decade have identified a large number of loci associated with increased risk of PD and helped prioritize genomic regions of interest for further functional characterization.

A major challenge for the Parkinson’s community is, therefore, to decipher the functional sequelae of the variants identified by GWA studies in order to achieve a deeper understanding of the genetic etiology of PD and uncover novel drug targets/pathways, thereby accelerating drug development. Parallel studies by a number of groups using different experimental approaches have investigated the functional roles of these variants such as their effects on gene expression (expression quantitative trait loci (eQTLs)) [4–7], long non-coding RNA trans-regulation [8], and protein-protein interaction networks [9] providing substantial insights into disease mechanisms for a number of common disorders. The impact of eQTLs is of particular interest as it can provide compelling evidence linking a risk variant and disease-specific genetic alterations in terms of altered expression and splicing levels, therefore yielding insight into the disease association and mechanism. Hence, an eQTL analytical approach can bridge the gap between the structural variants and their functional and regulatory implications which can facilitate further integrative analyses.

A number of eQTL studies have been conducted for different human diseases in order to understand the effect of the associated variants on the candidate transcripts. For example, prostate cancer risk SNPs were analyzed from 471 prostate tissues [10], identifying 51 significant eQTLs associated with 88 genes. In a subsequent study, an eQTL mapping approach was applied to human inflammatory bowel (IBD) diseases in five primary immune cell types. This study involved 91 patients with active inflammatory disease, 46 with antineutrophil cytoplasmic antibody-associated vasculitis, and 43 healthy controls. As a result, novel eQTLs in 34 IBD-associated loci were reported [11]. Other eQTL studies were performed at a genome wide scale in different control human tissues such as liver [12], blood and brain [13], and monocytes [14]. Outcomes of these studies highlighted the importance of tissue-specific eQTL and splicing QTLs in human disease.

In the context of brain disorders, a number of datasets are now publically available to look at gene expression on a regional and temporal basis [15, 16]. eQTL results from the Braineac dataset, which integrates whole genome genotype and transcript expression data from 134 human control brain samples of 10 brain regions [7, 17], allowing examination of genes implicated in PD by GWA analyses. Studies focusing on specific three PD loci, alpha synuclein (*SNCA*) [18], microtubule-associated protein tau (*MAPT*) [19], and leucine-rich repeat kinase (*LRRK2*) [4], were investigated separately in detail and have been published previously. In addition, targeted eQTL approaches have been applied in the context of PD by Latourelle and coworkers. The transcript expression profiling was performed on 23 PD prefrontal cortex brain cases and 24 controls in 5 GWAS-identified loci (*SNCA*, *MAPT*, *GAK/DGKQ*, *HLA*, and *RIT2*). The study identified multiple eQTLs which include both cis-acting SNP effects as well as trans-effects [20].

**Fig. 1:**
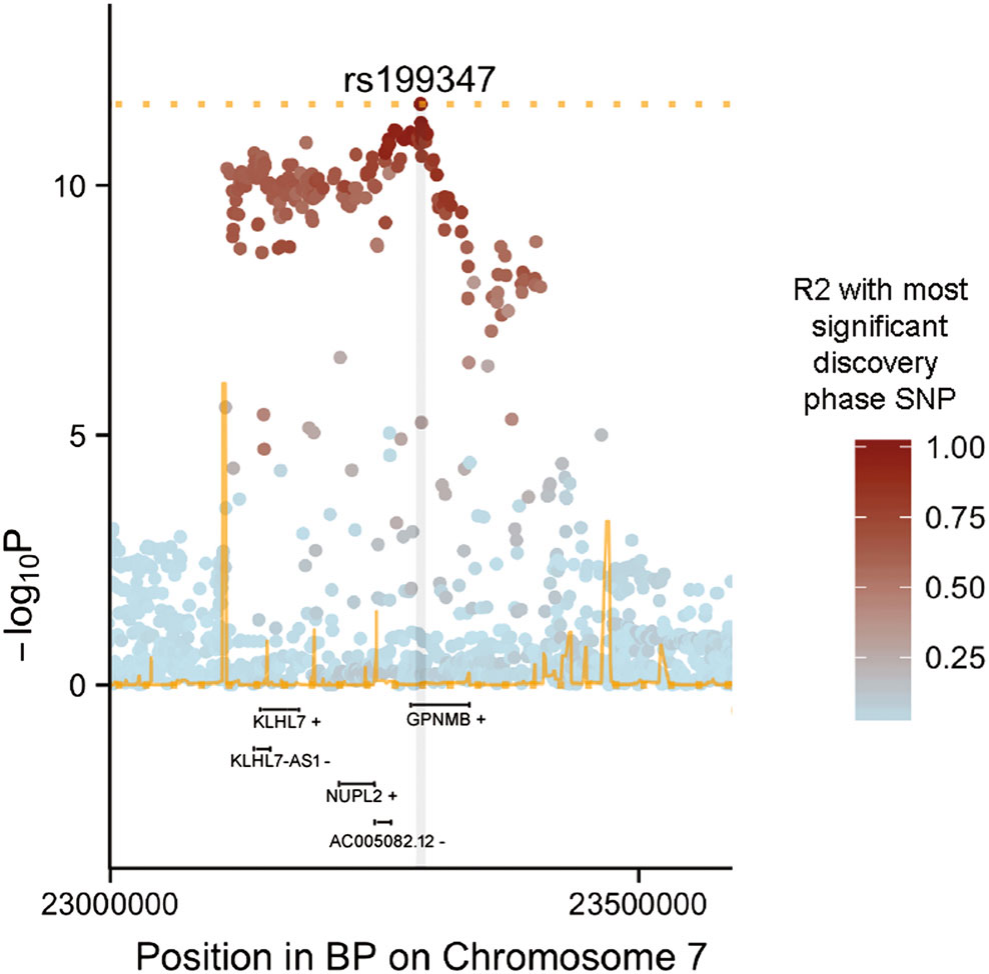
Regional association plot for rs199347 SNP at Chr7p15.3 locus from discovery phase. The plot shows regions ±1 Mb most significant SNP from PD GWAS study and the five transcripts in the locus investigated in this study. This locus was named as *GPNMB* locus. Only the five transcripts at this locus are shown in this figure. Figure modified from Nalls et al. [3]

In this current study, the hypothesis that PD GWA risk SNP rs199347 tagging the genomic location Chr7:23,145,089-23,314,256 bp (GRCh37) (Fig. 1) segregates as an eQTL with some or all of the transcripts at this genomic region. This hypothesis was tested by exploring all eQTLs of these five transcripts at this locus (*GPNMB*, *KLHL7*, *KLHL7-AS1*, *NUPL2*, and *AC005082.12*) using the Braineac microarray dataset [17]; a recent CAGEseq dataset [21]; GTEx Portal, which uses RNA sequence platform [22]; and NCBI’s Phenotype-Genotype Integrator (PheGenI) [6]. The performed analysis here does not cover epigenetic effect; however, it is a comprehensive analysis of reported GWAS signal (rs199347) in different brain tissues as well as other human tissues using multiple datasets including our in-house dataset (Braineac) and as a result, a step forward from Nalls et al. study [3].

## Results

In this study, we examined the functional effect of the PD risk SNP rs199347 on the mRNA expression levels of the transcripts at the Chr7p15.3 locus. This was performed by integrating expression data from different human brain tissues from Braineac, alongside other human tissues accessed via the GTEx portal through an eQTL approach (refer to Table 1 and Supplementary Table 1 for further details on the human tissues in GTEx) and an eQTL data generated from human frontal lobe tissues based on cap analysis gene expression sequencing (CAGEseq).

**Table 1:** rs199347 is a *GPNMB* eQTL specifically in the human brain tissues in Braineac, GTEx, CAGEseq, and PheGenI

GPNMB	Tissue specificity (brain)	No. of tissue	SNP (MOE)	*p* value	FDR
Braineac	Average across all 10 regions (aveALL)	134	rs199347 (+)	8.00E−13	5.10E−10
	Cerebellum (CRBL)			2.60E−06	7.57E−03
	Frontal cortex (FCTX) (BA 9,46)			3.70E−11	2.55E−08
	Hippocampus (HIPP)			5.20E−07	8.94E−05
	Medulla inferior olivary nucleus (MEDU)			2.00E−01	7.29E−01
	Occipital cortex (OCTX)			3.30E−09	2.34E−06
	Putamen (PUTM)			3.50E−08	3.22E−05
	Substantia nigra (SNIG)			4.90E−01	8.81E−01
	Temporal cortex (TCTX)			4.10E−11	1.60E−08
	Thalamus (THAL)			2.50E−03	2.72E−01
	Intralobular white matter (WHMT)			1.40E−02	4.51E−01
Total	10 tissues				
GTEx	Brain—anterior cingulate cortex (BA24)	72	rs199347 (+)		4.60E−10
	Brain—caudate (basal ganglia)	100			5.80E−13
	Brain—cerebellar hemisphere	89			2.50E−10
	Brain—cerebellum	103			3.20E−07
	Brain—cortex	96			1.70E−15
	Brain—frontal cortex (BA9)	92			1.30E−12
	Brain—hippocampus	81			1.10E−07
	Brain—hypothalamus	81			2.80E−09
	Brain—nucleus accumbens (basal ganglia)	93			2.10E−09
	Brain—putamen (basal ganglia)	82			2.00E−12
	Heart—atrial appendage	159			3.20E−13
	Heart—left ventricle	190			2.10E−17
	Prostate	87			1.20E−06
	Skin—sun exposed (lower leg)	302			7.70E−09
Total	14 tissues	1627			
PheGenI	Brain frontal cortex	143	rs199347 (+)	NA	7.58E−17
CAGEseq	Brain frontal cortex	119	rs199347 (+)	1.60E−11	4.97E−08

Table shows information extracted, summarized, and compared from Braineac, CAGEseq, GTEx, and PheGen datasets. The rs199347 is reported as a GWAS for PD, and it is a significant eQTL mostly in brain, specifically in cortical regions. It is clear that GPNMB eQTLs are brain specific followed by the heart and skin (for more details about other tissues and other SNPs, see Supplementary Table 1). Low numbers of less significant QTLs in other tissues are reported such as gastrointestinal tissues. No eQTLs were detected in other 21 human tissues that GTEx tested such as liver and kidney. Other reported SNPs are significant eQTLs in the three datasets, and they are in the same linkage disequilibrium (LD) with the SNP of interest rs199347. It is worth noting that different datasets reported same effect of rs199347 on GPNMB expression. MOE is the mode of effect. The (−) and (+) indicating the mode of the effect of the QTL on the expression either increase (+) or decrease (−) in association with the major allele. The *p* value is the unadjusted value of eQTL. False discovery rate (FDR) is the adjusted *p* value with FDR threshold 1%. The FDR was calculated within each tissue. Braineac and CAGEseq FDR threshold is 1%. GTEx and PheGen FDR threshold is 5% (for more details, please see Supplementary Tables 1 and 3)

**Fig. 2:**
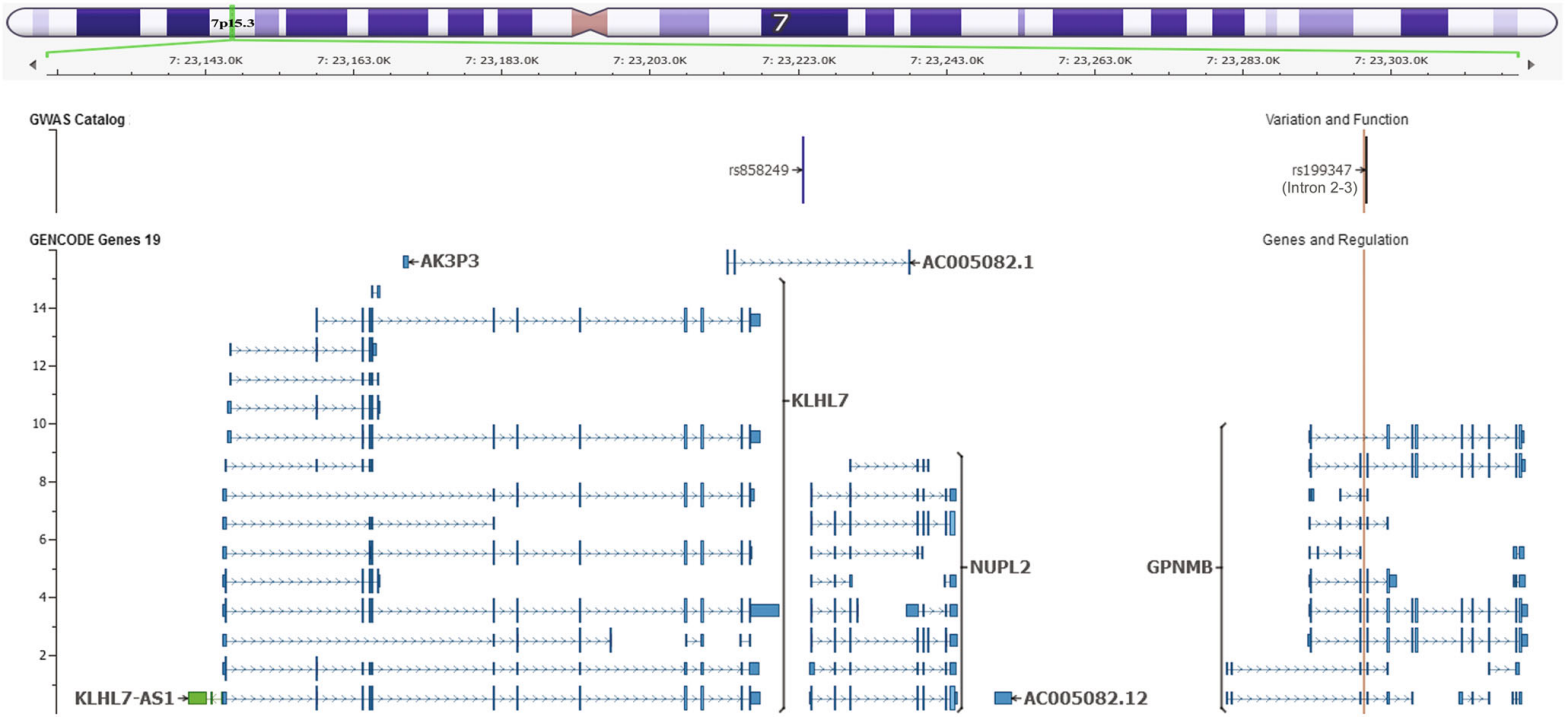
Genomic location of the Chr7p15.3 locus representing the five transcripts and SNP rs199347. The figure represents the genomic location of the genes in the Chr7p15.3 locus, namely, *GPNMB*, *KLHL7*, *KLHL7-AS1*, *NUPL2*, and *AC005082.12* along with their different isoforms*.* The position of rs199347 (intron 2–3 of *GPNMB*) is also represented. The GRCh37 build was used to construct the genomic location in this figure

**Fig. 3:**
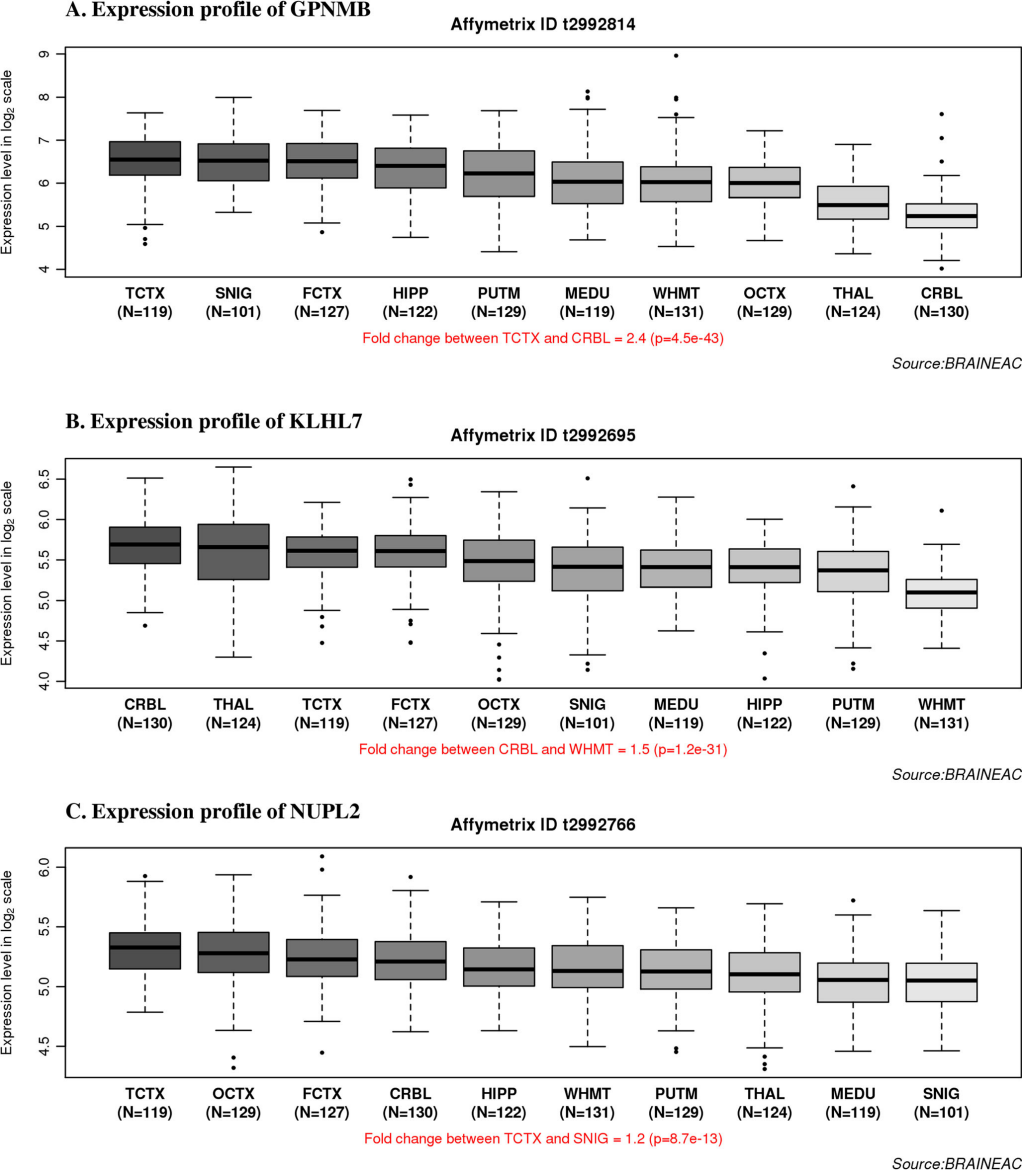
Regional distribution of mRNA expression patterns of the three transcripts (*GPNMB*, *KLHL7*, and *NUPL2*) in Chr7p15.3 locus in Braineac. **A** Box plot of mRNA expression levels for *GPNMB* in 10 brain regions, from microarray experiments on a log2 scale (*y* axis). *CRBL* cerebellum, *OCTX* occipital cortex, *FCTX* frontal cortex, *TCTX* temporal cortex, *SNIG* substantia nigra, *WHMT* white matter, *HIPP* hippocampus, *PUTM* putamen, *THAL* thalamus, *MEDU* medulla. This plot shows that GPNMB expression in TCTX is higher by 2.4-fold change (FC) compared with CRBL. **B** Box plot of mRNA expression levels for *KLHL7* in 10 brain regions, from microarray experiments on a log2 scale (*y* axis). This plot shows that *KLHL7* expression in CRBL is higher by 1.5 FC compared with WHMT. **C** Box plot of mRNA expression levels for *NUPL2* in 10 brain regions, from microarray experiments on a log2 scale (*y* axis). This plot shows that *NUPL2* expression in TCTX is higher by 1.2 FC compared with SNIG. *Whiskers* extend from the box to 1.5 times the inter-quartile range. *Whiskers* extend from the box to 1.5 times the inter-quartile range

Firstly, expression profiling for the five transcripts in the locus under consideration (*GPNMB*, *KLHL7*, *KLHL7-AS1*, *NUPL2*, and *AC005082.12*) (Fig. 1) were compared using Braineac and GTEx datasets (refer to Fig. 2 for the locus details). The expression profiles for the antisense *KLHL7*-*AS1*and long non-coding RNA (*AC005082.12*) species were attained from GTEx only as the microarray platform design does not cover long non-coding RNA species. The expression pattern of glycoprotein non-metastatic melanoma protein B (*GPNMB*) from the Braineac dataset revealed significant regional expression differences (2.4-fold change (FC), *p* = 4.5 × 10^−43^; refer to the “Materials and methods” section for further details) with TCTX showing the highest expression and cerebellum (CRBL) showing the lowest expression (Fig. 3a). The same pattern was confirmed from GTEx data showing lowest expression in CRBL and highest in cortical regions. Due to differences between the precise regions assessed in the different datasets, comparisons were performed among the most relevant matching brain region between the Braineac and GTEx datasets (Table 2). The expression level difference in the Kelch-like protein 7 (*KLHL7*) transcript observed in the Braineac dataset was a 1.5 FC with CRBL exhibiting the highest expression and white matter (WHMT) showing the lowest expression (*p* = 1.2 × 10^−31^; Fig. 3b), with similar pattern observed in the GTEx dataset showing a high expression in the cerebellar hemisphere in comparison with other brain regions. For nucleoporin-like protein 2 (*NUPL2*) transcript, a 1.2 FC with substantia nigra (SNIG) being the lowest and TCTX being the highest (*p* = 8.7 × 10^−13^) were observed (Fig. 3c) in Braineac. However, this was not the case in GTEx as CRBL showed the highest expression level followed by cortical regions. This can be understandable and we must allow expression variability, as it can be raised based on different platforms, dissection and extraction protocols, and quality controls between the two datasets.

**Table 2:** Brain regions from GTEx and relevant regions in Braineac

GTEx (brain regions)	Braineac
Anterior cingulate cortex (BA24)	NA
Amygdala	NA
Caudate (basal ganglia)	NA
Cerebellar hemisphere	Cerebellum (CRBL)
Cerebellum	Cerebellum (CRBL)
Cortex	Frontal cortex (FCTX)
	Occipital cortex (OCTX)
	Temporal cortex (TCTX)
Frontal cortex (BA9)	Frontal cortex (FCTX)
Hippocampus	Hippocampus (HIPP)
Hypothalamus	NA
Nucleus accumbens (basal ganglia)	NA
Substantia nigra	Substantia nigra (SNIG)
Spinal cord (cervical c-1)	NA
Putamen (basal ganglia)	Putamen (PUTM)

Table shows the different brain regions from GTEx (13 regions) and Braineac (10 regions) considered for comparison as not all brain regions in both studies overlap

**Fig. 4:**
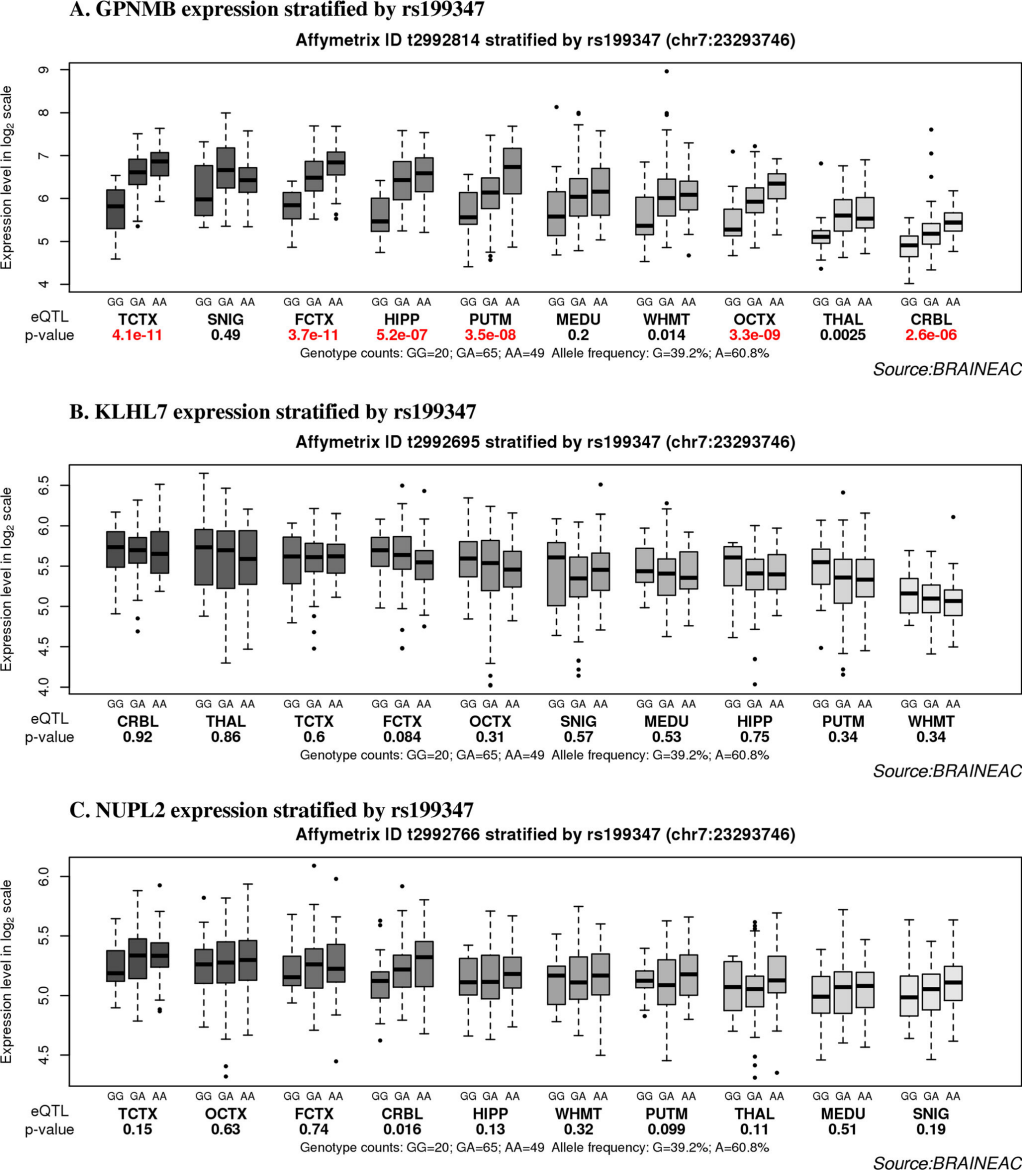
The effect of rs199347 on the expression levels of transcripts (*GPNMB*, *KLHL7*, and *NUPL2*) in Chr7p15.3 locus in Braineac. **A** Box plot shows *GPNMB* expression stratified by rs199347 in 134 brain samples. Increased expression was associated with the homozygous major allele (AA) in TCTX, FCTX, OCTX, HIPP, CRBL, and PUTM. A similar pattern was observed in other brain regions, but not as significantly. **B** Box plot shows *KLHL7* expression stratified by rs199347 in 134 brain samples. No significant association was observed after multiple testing correction FDR was applied. **C** Box plot shows *NUPL2* expression stratified by rs199347 in 134 brain samples. The SNP is associated with increased expression in CRBL, TCTX, and FCTX, although no significant association was observed after multiple testing correction FDR was applied. *Whiskers* extend from the box to 1.5 times the inter-quartile range

Secondly, the eQTL analyses for the PD risk SNP rs199347 in relation with the five transcripts were studied in detail using the abovementioned datasets. The investigation of rs199347, which is located in introns 2–3 of the *GPNMB* gene, showed significant eQTLs in several brain regions in all the four eQTL datasets, which are Braineac, CAGEseq, GTEx, and PheGenI. In Braineac, rs199347 was recorded as a significant eQTL with the *GPNMB* transcript (*p* = 8 × 10^−13^, average across all regions). The SNP is associated with increased mRNA expression with the major allele AA in temporal cortex (TCTX), frontal cortex (FCTX), hippocampus (HIPP), putamen (PUTM), occipital cortex (OCTX), and CRBL in normal individuals (Fig. 4a and Table 1). In the CAGEseq dataset, rs199347 was a significant eQTL in FCTX (*p* = 1.6 × 10^−11^) also for the major allele AA (Table 3 and Supplementary Table 3). In GTEx, rs199347 was also a significant eQTL in the brain (FCTX, caudate, HIPP, PUTM, and CRBL) and in heart and skin showing the same mode of effect (MOE) on the expression (+). In Table 1, further details and summary about eQTLs, mode of effect, and false discovery rate (FDR) values for *GPNMB* in the four eQTL datasets are shown. It is worth mentioning that several other eQTLs were found to be significant in *GPNMB*. For example, rs156425, rs6967526, and rs858272 were found to be significant in several brain regions across all datasets studied. The rs156425 was observed with the highest significance in FCTX, TCTX, OCTX, and PUTM, while rs6967526 and rs858272 showed high significance in FCTX, TCTX, OCTX, PUTM, and HIPP. The fact that the above SNPs belong to the same linkage disequilibrium (LD) as rs199347 justify their significance as eQTLs in the similar brain regions (refer to Table 1 for further details).

**Table 3:** rs199347 eQTLs for *KLHL7* and *NUPL2* in brain and other human tissues in GTEx, CAGEseq, and PheGenI

GTEx					
Gene name	SNP	No. of tissue	Tissue specificity	MOE	FDR
*KLHL7*	rs199347	93	Brain—nucleus accumbens (basal ganglia)	−	1.40E−06
		159	Heart—atrial appendage	+	2.40E−06
		190	Heart—left ventricle	+	3.70E−10
		361	Muscle—skeletal	+	2.50E−07
		278	Thyroid	−	5.90E−07
Total		1081	5 tissues		
*NUPL2*	rs199347	298	Adipose—subcutaneous	−	1.20E−19
		185	Adipose—visceral (omentum)	−	1.70E−10
		126	Adrenal gland	−	2.60E−09
		197	Artery—aorta	−	2.00E−07
		285	Artery—tibial	+	3.20E−06
		89	Brain—cerebellar hemisphere	+	8.30E−13
		103	Brain—cerebellum	+	1.10E−13
		96	Brain—cortex	+	4.90E−08
		93	Brain—nucleus accumbens (basal ganglia)	+	3.90E−06
		183	Breast—mammary tissue	+	1.50E−08
		272	Cells—transformed fibroblasts	+	2.40E−08
		169	Colon—transverse	+	3.50E−10
		127	Esophagus—gastroesophageal junction	+	4.40E−08
		218	Esophagus—muscularis	+	7.30E−07
		159	Heart—atrial appendage	+	2.70E−13
		190	Heart—left ventricle	+	1.20E−17
		278	Lung	+	1.10E−10
		361	Muscle—skeletal	+	4.20E−11
		256	Nerve—tibial	+	4.30E−17
		196	Skin—not Sun exposed (suprapubic)	+	1.80E−06
		302	Skin—Sun exposed (lower leg)	+	2.30E−06
		89	Spleen	−	2.40E−07
		278	Thyroid	+	7.40E−08
		338	Whole blood	+	4.90E−06
Total	4888	24 tissues			
PheGenI					
*NUPL2*	rs1474347	60	Lymphoblastoid	NA	1.08E−04
CAGEseq					
*NUPL2*	rs199347	119	Brain frontal cortex	+	3.59E−05

Table shows information extracted and summarized from GTEx, CAGEseq, and PheGenI datasets for rs199347 in association with the other transcripts in the GPNMB locus, *KLHL7* and *NUPL2*. The rs199347 is a significant eQTL with *KLHL7* in the basal ganglia in brain and thyroid causing decrease in the expression of the transcript with the major allele, while it is causing increase in the expression of the same transcript in heart and muscles. For *NUPL2*, this SNP is an eQTL in 24 tissues including adipose, heart, brain, muscle, and lung with highest significance association in CRBL. MOE is the mode of effect. The (−) and (+) indicate the mode of the effect of the QTL on the expression, either increase (+) or decrease (−) in association with the major allele. GTEx FDR threshold is 5% and CAGEseq FDR threshold is 1% (for more details, please see Supplementary Tables 2 and 3)

Thirdly, deeper analysis revealed that rs199347 SNP is associated not only with increased expression of the *GPNMB* transcript in brain but also with altered expression of four other transcripts at this locus, which are *KLHL7*, *KLHL7*-*AS1*, *NUPL2*, and *AC005082.12* (Tables 3 and 4 and Supplementary Table2). In GTEx, these transcripts demonstrated significant eQTL association resulting in decreased expression of *KLHL7* mRNA in thyroid and brain (nucleus accumbens) and increased expression in heart and skeletal muscle with FDR values ranging from 2.4 × 10^−06^ to 3.7 × 10^−10^ (Table 3 and Supplementary Table 2); although the similar expression effect was observed in Braineac, this eQTL did not pass the multiple correction FDR threshold (Fig. 4b). Most importantly, rs199347 shows the highest significant eQTL associations with *KLHL7*-*AS1* (antisense RNA1) and has decrease effect on the expression in 41 tissues (FDR ranging from 1 × 10^−13^ to 1 × 10^−39^) such as heart, lung, and many others. including brain regions (anterior cingulate cortex, HIPP, caudate, CRBL, FCTX, PUTM, and cortex) with less significant association (FDR ranging from 1 × 10^−06^ to 1 × 10^−13^) and increase effect on the expression in immune system tissue (spleen; FDR 3 × 10^−10^) (refer to Table 4 and Supplementary Table 2). In addition, rs199347 is associated with expression of a long non-coding RNA (*AC005082.12*) in tissues other than brain. This long non-coding RNA is located 29 kb 5′ to the *GPNMB* transcript (Fig. 2, Table 4, and Supplementary Table 2). It is important to mention that the expression profile and eQTL analyses for *KLHL7*-*AS1* and long non-coding RNA (*AC005082.12*) could not be obtained from Braineac as it is a microarray platform and the probes specific for this transcript were not included in the array design. Therefore, the comparison between GTEx and Braineac could not be performed.

**Table 4:** rs199347 eQTLs for *KLHL7-AS1* and *AC005082.12* in brain and other human tissues in GTEx

GTEx					
Gene name	SNP	No. of tissue	Tissue specificity	MOE	FDR
*KLHL7-AS1*	rs199347	72	Brain—anterior cingulate cortex (BA24)	−	1.10E−06
		100	Brain—caudate (basal ganglia)	−	2.90E−08
		89	Brain—cerebellar hemisphere	−	2.80E−07
		103	Brain—cerebellum	−	1.10E−08
		96	Brain—cortex	−	5.40E−13
		92	Brain—frontal cortex (BA9)	−	5.10E−08
		81	Brain—hippocampus	−	1.50E−06
		81	Brain—hypothalamus	−	1.30E−07
		93	Brain—nucleus accumbens (basal ganglia)	−	2.90E−07
		82	Brain—putamen (basal ganglia)	−	7.10E−08
		89	Spleen	+	3.00E−10
Total	978	11 tissues			
AC005082.12	rs199347	298	Adipose—subcutaneous	−	1.10E−14
		185	Adipose—visceral (omentum)	−	2.00E−12
		126	Adrenal gland	−	2.30E−07
		197	Artery—aorta	−	3.00E−07
		285	Artery—tibial	−	2.30E−08
		183	Breast—mammary tissue	−	1.70E−09
		124	Colon—sigmoid	−	1.30E−08
		127	Esophagus—gastroesophageal junction	−	1.50E−06
		241	Esophagus—mucosa	−	3.90E−11
		218	Esophagus—muscularis	−	3.60E−09
		159	Heart—atrial appendage	−	2.20E−10
		190	Heart—left ventricle	−	2.40E−11
		278	Lung	−	1.60E−07
		256	Nerve—tibial	−	3.70E−06
		196	Skin—not Sun exposed (suprapubic)	−	3.30E−07
		302	Skin—Sun exposed (lower leg)	−	5.40E−10
		278	Thyroid	+	1.10E−08
		338	Whole blood	−	6.80E−09
Total	3981	18 tissues			

Table shows the information extracted and summarized from GTEx datasets for rs199347 in association with the other transcripts in the GPNMB locus, *KLHL7-AS1* and *AC005082.12*. The rs199347 is a significant eQTL with *KLHL7-AS1* in the brain (cortex, PUTM, and HIPP) and other 33 human tissues with higher significant FDR values. The SNP has the same effect in all brain regions by decreasing the expression of the transcript with the major allele. However, there is opposite effect in the spleen. The same effect on the expression of the long non-coding RNA *AC005082.12* is also observed in 18 human tissues but not the brain. MOE is the mode of effect. The (−) and (+) indicating the mode of the effect of the QTL on the expression either increase (+) or decrease (−) in association with the major allele. GTEx FDR threshold is 5% (for more details, please see Supplementary Table 2)

Finally, the SNP rs199347 is an eQTL with *NUPL2* transcript and shows an increased expression in 24 tissues including brain, heart, lung, spleen, and skin (FDR ranging from 4.9 × 10^−06^ to1.2 × 10^−19^) in GTEx (Table 3 and Supplementary Table 2). Similar pattern of increasing mRNA expression of *NUPL2* in relation to rs199347 was also observed in the FCTX, CRBL, and PUTM in Braineac, but it did not pass the multiple test FDR correction (Fig. 4c). In the CAGEseq dataset, rs199347 was a significant eQTL in FCTX (FDR = 3.6 × 10^−05^) also for the major allele AA (Table 3 and Supplementary Table 3). An additional eQTL (rs1474347) was revealed in lymphoblastoid cells with *NUPL2* with FDR 1 × 10^−04^.

## Discussion

Over the past two decades, GWA studies have revolutionized our understanding of common genetic variation and helped us to map genomic loci that are associated with increased risk for common human disease. The majority of these risk variants, however, are not associated with coding changes in expressed proteins [23], and a major challenge for the research community is to identify and understand the subtle functional consequences of non-coding genetic variation linked to disease risk in the human genome. These functional modifications can be via altered expression, splicing, and methylation patterns of targeted transcripts and proteins that can be localized in specific tissues, regions or cells, and at specific time points in development or ageing. GWA data provides, therefore, only the starting point in terms of understanding the functional impact on transcripts and proteins in the context of disease etiology. One approach to achieve greater understanding of the link between genomic variation and functional consequence is to combine GWAS and multiple eQTL studies to understand the functional effects of risk loci and provide further information about the link between genetic association and cellular mechanisms [24]. Previous analysis of eQTL results from the Braineac resource revealed that 17.4% of GWAS SNPs associated with brain-related traits were functional eQTLs [7]. A number of other studies have used same approach by overlapping GWAS and whole genome eQTL results for different human diseases to prioritize targeted loci/transcripts for further biological experiments [2, 10, 21].

Applying an eQTL analysis approach can shed light on variation in gene expression associated with PD and help to develop our understanding of disease etiology. Both Braineac and GTEx gene expression datasets revealed differential expression levels between different brain regions (Braineac) and other human tissues (GTEx) for the named transcripts at the chromosome 7 PD association locus. The data presented above demonstrate that the risk SNP rs199347 is an eQTL with the five transcripts we investigated at this locus (*GPNMB*, *KLHL7*, *KLHL7-AS1*, *NUPL2*, and *AC005082.12*) at different significant levels in different brain regions, cortex, and PUTM being the highest, as well as in other human tissues such as heart and skin. It is important to note that this genomic locus on Chr7p15.3 (∼169 kb) is in high LD block structure based on the HapMap project [25], a fact further emphasized by the spread of genome wide significant SNPs identified in the PD GWAS and displayed in Fig. 1. It is therefore challenging to dissect and specify from which gene/transcript the signal is driven, although the increase in the expression of *GPNMB* in multiple datasets (Braineac, CAGEseq, and GTEx), and the localization of the most significant risk SNP at the locus to the *GPNMB* gene, suggests that the *GPNMB* is the most logical candidate coding gene in the Chr7p15.3 locus. These data, however, do not exclude potentially important functional roles for the other transcripts, antisense, and sense non-coding RNA species within this locus. *GPNMB* revealed brain-specific eQTLs in most brain regions, which are reported and confirmed in both datasets (refer to Table 1). In addition, based on a recent study that identified PD risk loci that linked to immune system relevant to PD [23], no eQTLs were observed in any immune system tissues (e.g., spleen and lymphoblastoid cells) in all three datasets for this transcript. For *NUPL2* and *KLHL7*, only eQTLs in brain and other human tissues from CAGEseq and GTEx passed the FDR threshold (refer to Table 3). *KLHL7-AS1* revealed the most significant eQTLs in brain tissues in the GTEx dataset (refer to Table 4). It is worth noting that the *KLHL7* transcript demonstrates significant eQTL in only 5 out of 44 tissues in GTEx, while *KLHL7-AS1* shows significant associations with the risk SNP in 43 tissues with an opposite effect on the expression. This supports a role for the antisense RNA as a key regulator of *KLHL7* in diverse tissues and demands more consideration in future studies to understand its interaction with other transcripts in greater detail. In addition, expression of the long non-coding RNA *AC005082.12* was increased in a range of human tissues (although notably not brain tissue) as an eQTL associated with rs199347 (refer to Table 4). Interestingly, rs199347 eQTL shows differences in its effects on the mRNA expression patterns in the brain tissues, as it shows higher expression with the major allele in case of *GPNMB*, *KLHL7*, and *NUPL2*, but shows decreasing in the expression of *KLHL7-AS1*. The data reported by Nalls et al. indicated that the rs199347 is associated with increased expression of *NUPL2* and decreased methylation of *GPNMB* in FCTX and CRBL brain regions [3]. Previous studies compared single-cell-type specific expression patterns for human *GPNMB* in the mouse astrocytes, neurons, OPC, oligodendrocytes, microglia, and endothelial tissues, demonstrating that *GPNMB* is highly expressed in glial cell populations, while the expression in neurons is minimal. This calls for further human single-cell expression studies (Supplementary Fig. 1) [26], which would aid in building on the existing knowledge regarding cell-specific functional mechanisms in PD. This suggests a complex role of the eQTL that could be transcript, tissue, cell specific, and species and demands further investigations on possible functional interaction between these coding transcripts and antisense and sense non-coding RNA species in the brain.

A confounding factor when interpreting and understanding genome wide association data is that reported associations can be skewed by population-specific aspects of the results. It is of note that a GWAS conducted in PD, amyotrophic lateral sclerosis (ALS), and multiple system atrophy (MSA) cohorts from a Han Chinese population reported that *GPNMB* has no association of rs156429, which is located in intron 6–7 of *GPNMB* gene, Chr7:23,266,401, with PD; this SNP is in strong LD with rs199347, suggesting that either the association is population specific or indicating the need of meta-analyses in large cohorts in order to eradicate false negative results [27].

In terms of biological roles, GPNMB is a glycoprotein transmembrane protein of unknown function. It has been reported to have a potential neuroprotective role in the spinal cord of an ALS mouse model and showed high protein expression level in CSF of human ALS patients [28]. Intriguingly, *GPNMB* mRNA and protein expression have also been linked to Gaucher’s disease [29] and Niemann Pick type C [30], two lysosomal storage disorders. The former has important genetic links with PD, reinforcing a potential link between this protein and PD. Equally of interest is a role for *GPNMB* in the severity of IBD models [31], as other PD link genes (notably *LRRK2*) have demonstrated a phenotypic overlap with IBD [4]. A number of studies have linked increased expression of *GPNMB* to tumors, and indeed, *GPNMB* is being used as a potential binding partner for targeting drugs to cancerous cells [28, 32]. Gene ontology suggests that the *GPNMB* plays a role in many molecular functions, for example, integrin binding, protein complex binding, ion binding, and receptor binding. *NUPL2* is part of nuclear export signal receptor, mRNA transport, and establishment of RNA localization. *KLHL7* acts as a mediator for protein ubiquitination and modification [33, 34]. Further biological investigation relating to the role of these genes in cellular pathways and function is vital and could clarify a putative role for one of these genes in association with the eQTL in PD.

In summary, the results of this study reinforce a need for greater functional characterization of the biological roles of the genes at this locus in order to determine their potential role in the etiology of PD, with GPNMB prioritized for such treatment. These data also further emphasize the challenges presented by the GWA analyses with regard to developing a detailed mechanistic understanding of pathways to disease and highlights the importance of combining genetic approaches with functional analysis and investigations to improve resolution of these issues. The data presented herein suggests that an increased expression of *GPNMB* in brain tissue underlies the association between PD risk and chromosome 7p15.3. With currently available datasets and analysis techniques, however, it is not possible to exclude alterations in other genes at the locus as the causative link between 7p15.3 and PD. Further experimental investigation into gene expression and functional variation at this locus is, therefore, a priority.

## Materials and methods

### Expression and eQTL analysis

Transcripts within Chr7p15.3 locus (genomic location Chr7:23,145,089–23,314,256 bp, ∼169 kb, GRCh37) tagged by the risk SNP rs199347 in PD GWAS study [3] were taken as potential candidates for expression (eQTL) analysis.

eQTL reporting and analysis were performed on several datasets including the in-house dataset, Braineac, which contains Brain tissues originating from 134 control individuals collected by the Medical Research Council (MRC) Sudden Death Brain and Tissue Bank, Edinburgh. The dataset contains brain tissues from the following regions: FCTX Brodmann areas 9 and 46; TCTX Brodmann areas 21, 41, and 42; parietal parasaggital (PCTX) Brodmann areas 3, 1, and 2; OCTX (specifically primary visual cortex) Brodmann area 17; HIPP; thalamus (THAL); PUTM; SNIG; medulla (MEDU; specifically inferior olivary nucleus); CRBL; and intralobular WHMT below Brodmann areas 39 and 40. RNA isolation and processing of brain samples were performed and analyzed using Affymetrix Exon 1.0 ST Arrays. In parallel, genomic DNA was extracted and gentotyped on the Illumina Infinium Human Omni1-Quad BeadChip. The QTL analysis was run for each expression profile (either exon level or transcript level) against every genetic marker (either SNP or indel) in Matrix eQTL [35]. Subsequent analyses were conducted in R open source software. A detailed description of the samples used in the study, tissue processing, dissection, and analysis pipeline is provided in main published papers for Braineac dataset [4, 7, 17]. ANOVA modules (method of moments) were performed using Partek® Genomics Suite^™^ to determine differentially expressed transcripts among 10 regions. The date of array hybridization (batch effects), gender, region, and individual were included as covariates to eliminate the possibility of variability that influences the expression profiles. All *p* values were corrected for multiple comparisons using the FDR step-up method. The eQTL results were classified by the marker type, SNP or indel; expression type, exon or gene/transcript level; and the distance of SNP to the transcription start site, cis or trans. Then, the FDR was calculated by Matrix eQTL [35] based on the Benjamin-Hochberg method. Basically, it takes into account the multiple tests performed based on a single probe, which includes all the SNPs around 1 Mb window of the boundaries of the probe. Only the associations with FDR <1% were considered for the subsequent analyses. All data is now publicly available online at http://www.braineac.org/. The eQTLs obtained for the transcripts in the Chr7p15.3 locus in Braineac dataset were cross verified in multiple datasets from the GTEx portal and NCBI’s PheGenI in brain and different tissues. All the QTL data were downloaded, collected, and summarized in Table 1 and Supplementary Table 1 based on the most significant SNP as eQTL and tissue specificity.

GTEx dataset [36] consists of a total of 8555 samples from 53 tissues of 544 donors for which RNAseq was conducted. The dataset has eQTL analysis for 7051 samples from 44 tissues of 449 individuals which combine genotype data from whole exome and genome sequencing as well as expression data from microarray and RNA sequencing. eQTL analysis was performed using Matrix eQTL [35]. FDR of 5% threshold was used to correct for multiple hypothesis. Data is available from the publicly available database at http://www.gtexportal.org/home/. Data was downloaded on July 2016, version 6. It is noteworthy that not all the brain regions in Braineac and GTEx datasets directly overlap. In these cases, the most relevant and closest region was taken for comparison. See Table 2 for more details.

PheGenI merges the NHGRI-GWAS catalogue data with several databases at NCBI, including Gene, dbGaP, OMIM, GTEx, and dbSNP. The eQTL data consists of 1269 samples from 7 tissues. The data is available at NCBI’s PheGenI website (http://www.ncbi.nlm.nih.gov/gap/phegeni) or in the eQTL browser (https://www.ncbi.nlm.nih.gov/projects/gap/eqtl/index.cgi). CAGEseq data was obtained from a previous published study consisting 119 FCTX samples. eQTL analysis was performed using Matrix eQTL with covariate postmortem interval, age, gender, and RNA integrity number and the first six principal components. A detailed description of the included samples, library preparation, and analysis pipeline is provided in main published paper [21].

### Expression in single-cell types of human and mouse brain tissues

The expression pattern for the transcripts was studied for eight single-cell types, namely, neurons, astrocytes, oligodendrocyte precursor cells, newly formed oligodendrocytes, myelinating oligodendrocytes, microglia, and endothelial cells from the database-RNA sequence transcriptome and splicing database of glia, neurons, and vascular cells of the cerebral cortex [26]. The data is publicly available at http://web.stanford.edu/group/barres_lab/brainseqMariko/brainseq2.html.

## Electronic supplementary material

Supplementary Figure 1 mRNA expression of GPNMB in cell specific type (astrocytes, neurons, oligodendrocyte precursor cells (OPC), oligodendrocytes, microglia/macrophage and endothelial) of mouse and human cerebral cortex. Expression level estimation was reported as fragments per kilo base of transcript sequence per million mapped fragments (FPKM) value. Differential expression was calculated as the FPKM of a given cell type divided by the average FPKM of all other cell types. (A) Specific cell type of mRNA expression from mouse cortex. Figure shows variability in GPNMB expression in different cell types, with the highest expression in microglia/macrophages and OPC compare to astrocytes, neurons and oligodendrocyte showing the lowest expression. (B) Specific cell type expression from human brain cells. Figure shows different cell specific variabilities in GPNMB expression in human compare with mouse. Microglia/macrophages and oligodendrocyte show higher expression in comparison with astrocytes and neurons cells. Figure is adapted from [26]. (GIF 315 kb)

High Resolution Image (TIFF 1104 kb)

Supplementary Table 1 (excel sheet attached), this table is an extended and more detailed form of the main table (Table 1). Table shows an example of most significant GPNMB QTLs in different tissues and datasets. Detailed information was extracted, summarized and compared from 3 datasets (Braineac, GTEx and PheGen). In addition, PD GWAS SNP rs199347 was checked and reported. This SNP is a significant eQTL mostly in human brain, specifically in cortical regions. Low numbers of less significant QTLs in other tissues are reported such as, gastrointestinal tissues. No eQTLs were detected in other 21 human tissues that GTEx tested such as liver and kidney. Green highlighted SNPs are significant in all datasets and in the same linkage disequilibrium (LD) region of the SNP of interest (rs199347). It is worth noting that different datasets reported same effect of rs199347 on GPNMB expression. The (−) and (+) indicate the mode of the effect of the QTL on the expression, either increase (+) or decrease (−) in association with major allele. The MOE column shows the majority of individuals with a dominant mode of effect on the transcript expression, but in our case it is GPNMB. Furthermore, the most significant reported SNP in the table is presenting the major effect in more individuals. *P-value* is the unadjusted *p-value* of eQTL. FDR is the adjusted *p-value* with FDR threshold 1%. The FDR was calculated within each tissue. Braineac FDR threshold is 1%. GTEx and PheGenI FDR threshold is 5%. (XLSX 27 kb)

Supplementary Table 2 (excel sheet attached), this table is an extended form of Tables 2 and 3. Table shows rs199347 QTLs with *GPNMB*, *KLHL7* and *NUPL2* in Braineac, in addition with *KLHL7-AS1* and *AC005082.12* in GTEx. This SNP is a significant eQTL in human brain and other tissues. FDR is the adjusted *p-value* with FDR threshold 1%. The FDR was calculated within each tissue. Braineac FDR threshold is 1%. GTEx FDR threshold is 5%. (XLSX 22 kb)

Supplementary Table 3 (excel sheet attached), this table shows all identified CAGEseq *cis* eQTLs for *GPNMB* and *NUPL2* transcripts including the PD risk SNP rs199347 and other SNPs in the same region (LD). CAGEseq FDR threshold is 1%. Information about the variants rs number, chromosome number, *p*-values, FDR and eQTLs statistic are provided in the table. (XLSX 105 kb)

## References

[1] Lees AJ, Hardy J, Revesz T (2009). Parkinson’s disease. Lancet.

[2] Westra HJ, Franke L (2014). From genome to function by studying eQTLs. Biochim Biophys Acta.

[3] Nalls MA, Pankratz N, Lill CM, Do CB, Hernandez DG, Saad M, DeStefano AL, Kara E, Bras J, Sharma M, Schulte C, Keller MF, Arepalli S, Letson C, Edsall C, Stefansson H, Liu X, Pliner H, Lee JH, Cheng R, Ikram MA, Ioannidis JP, Hadjigeorgiou GM, Bis JC, Martinez M, Perlmutter JS, Goate A, Marder K, Fiske B, Sutherland M, Xiromerisiou G, Myers RH, Clark LN, Stefansson K, Hardy JA, Heutink P, Chen H, Wood NW, Houlden H, Payami H, Brice A, Scott WK, Gasser T, Bertram L, Eriksson N, Foroud T, Singleton AB, International Parkinson’s Disease Genomics C, Parkinson’s Study Group Parkinson’s Research: The Organized GI, andMe, GenePd, NeuroGenetics Research C, Hussman Institute of Human G, Ashkenazi Jewish Dataset I, Cohorts for H, Aging Research in Genetic E, North American Brain Expression C, United Kingdom Brain Expression C, Greek Parkinson’s Disease C, Alzheimer Genetic Analysis G (2014). Large-scale meta-analysis of genome-wide association data identifies six new risk loci for Parkinson’s disease. Nat Genet.

[4] Trabzuni D, Ryten M, Emmett W, Ramasamy A, Lackner KJ, Zeller T, Walker R, Smith C, Lewis PA, Mamais A, de Silva R, Vandrovcova J, Hernandez D, Nalls MA, Sharma M, Garnier S, Lesage S, Simon-Sanchez J, Gasser T, Heutink P, Brice A, Singleton A, Cai H, Schadt E, Wood NW, Bandopadhyay R, Weale ME, Hardy J, Plagnol V, International Parkinson Disease Genomics C (2013). Fine-mapping, gene expression and splicing analysis of the disease associated LRRK2 locus. PLoS One.

[5] Yu CH, Pal LR, Moult J (2016). Consensus genome-wide expression quantitative trait loci and their relationship with human complex trait disease. OMICS.

[6] Gibbs JR, van der Brug MP, Hernandez DG, Traynor BJ, Nalls MA, Lai SL, Arepalli S, Dillman A, Rafferty IP, Troncoso J, Johnson R, Zielke HR, Ferrucci L, Longo DL, Cookson MR, Singleton AB (2010). Abundant quantitative trait loci exist for DNA methylation and gene expression in human brain. PLoS Genet.

[7] Ramasamy A, Trabzuni D, Guelfi S, Varghese V, Smith C, Walker R, De T, Coin L, de Silva R, Cookson MR, Singleton AB, Hardy J, Ryten M, Weale ME, Consortium UKBE, North American Brain Expression C (2014). Genetic variability in the regulation of gene expression in ten regions of the human brain. Nat Neurosci.

[8] Yin J, Wen J, Hang D, Han J, Jiang J, Song C, Liu Y, Liu J, Liu L, Zhu L, Chen J, Zhai X, Xie S, Hu Z, Shen H, Dai M, Li N (2015). Expression quantitative trait loci for CARD8 contributes to risk of two infection-related cancers—hepatocellular carcinoma and cervical cancer. PLoS One.

[9] Suthram S, Beyer A, Karp RM, Eldar Y, Ideker T (2008). eQED: an efficient method for interpreting eQTL associations using protein networks. Mol Syst Biol.

[10] Thibodeau SN, French AJ, McDonnell SK, Cheville J, Middha S, Tillmans L, Riska S, Baheti S, Larson MC, Fogarty Z, Zhang Y, Larson N, Nair A, O’Brien D, Wang L, Schaid DJ (2015). Identification of candidate genes for prostate cancer-risk SNPs utilizing a normal prostate tissue eQTL data set. Nat Commun.

[11] Peters JE, Lyons PA, Lee JC, Richard AC, Fortune MD, Newcombe PJ, Richardson S, Smith KG (2016). Insight into genotype-phenotype associations through eQTL mapping in multiple cell types in health and immune-mediated disease. PLoS Genet.

[12] Schadt EE, Molony C, Chudin E, Hao K, Yang X, Lum PY, Kasarskis A, Zhang B, Wang S, Suver C, Zhu J, Millstein J, Sieberts S, Lamb J, GuhaThakurta D, Derry J, Storey JD, Avila-Campillo I, Kruger MJ, Johnson JM, Rohl CA, van Nas A, Mehrabian M, Drake TA, Lusis AJ, Smith RC, Guengerich FP, Strom SC, Schuetz E, Rushmore TH, Ulrich R (2008). Mapping the genetic architecture of gene expression in human liver. PLoS Biol.

[13] Heinzen EL, Ge D, Cronin KD, Maia JM, Shianna KV, Gabriel WN, Welsh-Bohmer KA, Hulette CM, Denny TN, Goldstein DB (2008). Tissue-specific genetic control of splicing: implications for the study of complex traits. PLoS Biol.

[14] Zeller T, Wild P, Szymczak S, Rotival M, Schillert A, Castagne R, Maouche S, Germain M, Lackner K, Rossmann H, Eleftheriadis M, Sinning CR, Schnabel RB, Lubos E, Mennerich D, Rust W, Perret C, Proust C, Nicaud V, Loscalzo J, Hubner N, Tregouet D, Munzel T, Ziegler A, Tiret L, Blankenberg S, Cambien F (2010). Genetics and beyond—the transcriptome of human monocytes and disease susceptibility. PLoS One.

[15] Myers AJ, Gibbs JR, Webster JA, Rohrer K, Zhao A, Marlowe L, Kaleem M, Leung D, Bryden L, Nath P, Zismann VL, Joshipura K, Huentelman MJ, Hu-Lince D, Coon KD, Craig DW, Pearson JV, Holmans P, Heward CB, Reiman EM, Stephan D, Hardy J (2007). A survey of genetic human cortical gene expression. Nat Genet.

[16] Kang HJ, Kawasawa YI, Cheng F, Zhu Y, Xu X, Li M, Sousa AM, Pletikos M, Meyer KA, Sedmak G, Guennel T, Shin Y, Johnson MB, Krsnik Z, Mayer S, Fertuzinhos S, Umlauf S, Lisgo SN, Vortmeyer A, Weinberger DR, Mane S, Hyde TM, Huttner A, Reimers M, Kleinman JE, Sestan N (2011). Spatio-temporal transcriptome of the human brain. Nature.

[17] Trabzuni D, Ryten M, Walker R, Smith C, Imran S, Ramasamy A, Weale ME, Hardy J (2011). Quality control parameters on a large dataset of regionally dissected human control brains for whole genome expression studies. J Neurochem.

[18] Simon-Sanchez J, Schulte C, Bras JM, Sharma M, Gibbs JR, Berg D, Paisan-Ruiz C, Lichtner P, Scholz SW, Hernandez DG, Kruger R, Federoff M, Klein C, Goate A, Perlmutter J, Bonin M, Nalls MA, Illig T, Gieger C, Houlden H, Steffens M, Okun MS, Racette BA, Cookson MR, Foote KD, Fernandez HH, Traynor BJ, Schreiber S, Arepalli S, Zonozi R, Gwinn K, van der Brug M, Lopez G, Chanock SJ, Schatzkin A, Park Y, Hollenbeck A, Gao J, Huang X, Wood NW, Lorenz D, Deuschl G, Chen H, Riess O, Hardy JA, Singleton AB, Gasser T (2009). Genome-wide association study reveals genetic risk underlying Parkinson’s disease. Nat Genet.

[19] Trabzuni D, Wray S, Vandrovcova J, Ramasamy A, Walker R, Smith C, Luk C, Gibbs JR, Dillman A, Hernandez DG, Arepalli S, Singleton AB, Cookson MR, Pittman AM, de Silva R, Weale ME, Hardy J, Ryten M (2012). MAPT expression and splicing is differentially regulated by brain region: relation to genotype and implication for tauopathies. Hum Mol Genet.

[20] Latourelle JC, Dumitriu A, Hadzi TC, Beach TG, Myers RH (2012). Evaluation of Parkinson disease risk variants as expression-QTLs. PLoS One.

[21] Blauwendraat C, Francescatto M, Gibbs JR, Jansen IE, Simon-Sanchez J, Hernandez DG, Dillman AA, Singleton AB, Cookson MR, Rizzu P, Heutink P (2016). Comprehensive promoter level expression quantitative trait loci analysis of the human frontal lobe. Genome Med.

[22] Carithers LJ, Ardlie K, Barcus M, Branton PA, Britton A, Buia SA, Compton CC, DeLuca DS, Peter-Demchok J, Gelfand ET, Guan P, Korzeniewski GE, Lockhart NC, Rabiner CA, Rao AK, Robinson KL, Roche NV, Sawyer SJ, Segre AV, Shive CE, Smith AM, Sobin LH, Undale AH, Valentino KM, Vaught J, Young TR, Moore HM, Consortium GT (2015). A novel approach to high-quality postmortem tissue procurement: the GTEx project. Biopreserv Biobank.

[23] Coetzee SG, Pierce S, Brundin P, Brundin L, Hazelett DJ, Coetzee GA (2016). Enrichment of risk SNPs in regulatory regions implicate diverse tissues in Parkinson’s disease etiology. Sci Rep.

[24] Montgomery SB, Dermitzakis ET (2011). From expression QTLs to personalized transcriptomics. Nat Rev Genet.

[25] International HapMap C (2003). The International HapMap project. Nature.

[26] Zhang Y, Chen K, Sloan SA, Bennett ML, Scholze AR, O’Keeffe S, Phatnani HP, Guarnieri P, Caneda C, Ruderisch N, Deng S, Liddelow SA, Zhang C, Daneman R, Maniatis T, Barres BA, Wu JQ (2014). An RNA-sequencing transcriptome and splicing database of glia, neurons, and vascular cells of the cerebral cortex. J Neurosci.

[27] Xu Y, Chen Y, Ou R, Wei QQ, Cao B, Chen K, Shang HF (2016). No association of GPNMB rs156429 polymorphism with Parkinson’s disease, amyotrophic lateral sclerosis and multiple system atrophy in Chinese population. Neurosci Lett.

[28] Tanaka H, Shimazawa M, Kimura M, Takata M, Tsuruma K, Yamada M, Takahashi H, Hozumi I, Niwa J, Iguchi Y, Nikawa T, Sobue G, Inuzuka T, Hara H (2012). The potential of GPNMB as novel neuroprotective factor in amyotrophic lateral sclerosis. Sci Rep.

[29] Kramer G, Wegdam W, Donker-Koopman W, Ottenhoff R, Gaspar P, Verhoek M, Nelson J, Gabriel T, Kallemeijn W, Boot RG, Laman JD, Vissers JP, Cox T, Pavlova E, Moran MT, Aerts JM, van Eijk M (2016). Elevation of glycoprotein nonmetastatic melanoma protein B in type 1 Gaucher disease patients and mouse models. FEBS Open Bio.

[30] Marques AR, Gabriel TL, Aten J, van Roomen CP, Ottenhoff R, Claessen N, Alfonso P, Irun P, Giraldo P, Aerts JM, van Eijk M (2016). Gpnmb is a potential marker for the visceral pathology in Niemann-pick type C disease. PLoS One.

[31] Sasaki F, Kumagai K, Uto H, Takami Y, Kure T, Tabu K, Nasu Y, Hashimoto S, Kanmura S, Numata M, Moriuchi A, Sakiyama T, Tsubouchi H, Ido A (2015). Expression of glycoprotein nonmetastatic melanoma protein B in macrophages infiltrating injured mucosa is associated with the severity of experimental colitis in mice. Mol Med Rep.

[32] Zhou LT, Liu FY, Li Y, Peng YM, Liu YH, Li J (2012). Gpnmb/osteoactivin, an attractive target in cancer immunotherapy. Neoplasma.

[33] Wang J, Duncan D, Shi Z, Zhang B (2013) WEB-based GEne SeT AnaLysis Toolkit (WebGestalt): update 2013. Nucleic Acids Res 41 (Web Server issue):W77–83. doi:10.1093/nar/gkt439 10.1093/nar/gkt439 PMC3692109 23703215

[34] Zhang B, Kirov S, Snoddy J (2005) WebGestalt: an integrated system for exploring gene sets in various biological contexts. Nucleic acids res 33 (web server issue):W741-748. doi:10.1093/nar/gki475 10.1093/nar/gki475 PMC1160236 15980575

[35] Shabalin AA (2012). Matrix eQTL: ultra fast eQTL analysis via large matrix operations. Bioinformatics.

[36] Consortium GT (2013). The genotype-tissue expression (GTEx) project. Nat Genet.

