# Supplementary figures and images for: Increased brain expression of GPNMB is associated with genome wide significant risk for Parkinson’s disease on chromosome 7p15.3
Source: Neurogenetics. 2017 Apr 8;18(3):121–33. doi: 10.1007/s10048-017-0514-8 (PMC5522530; doi:10.1007/s10048-017-0514-8)

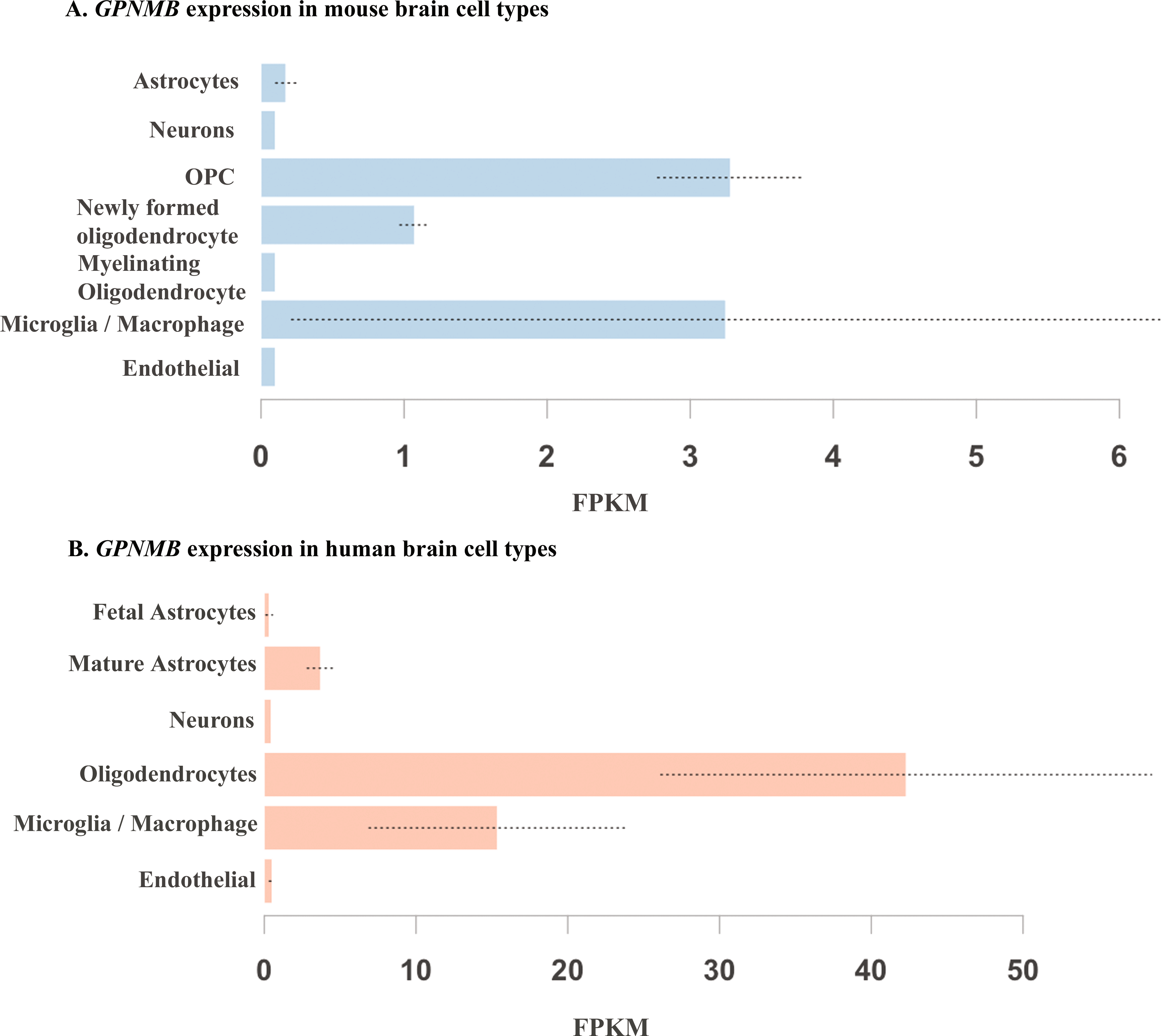

Supplement: Supplementary file 1 — mRNA expression of GPNMB in cell specific type (astrocytes, neurons, oligodendrocyte precursor cells (OPC), oligodendrocytes, microglia/macrophage and endothelial) of mouse and human cerebral cortex. Expression level estimation was reported as fragments per kilo base of transcript sequence per million mapped fragments (FPKM) value. Differential expression was calculated as the FPKM of a given cell type divided by the average FPKM of all other cell types. (A) Specific cell type of mRNA expression from mouse cortex. Figure shows variability in GPNMB expression in different cell types, with the highest expression in microglia/macrophages and OPC compare to astrocytes, neurons and oligodendrocyte showing the lowest expression. (B) Specific cell type expression from human brain cells. Figure shows different cell specific variabilities in GPNMB expression in human compare with mouse. Microglia/macrophages and oligodendrocyte show higher expression in comparison with astrocytes and neurons cells. Figure is adapted from [26]. (GIF 315 kb) [file 10048_2017_514_Fig5_ESM.gif]

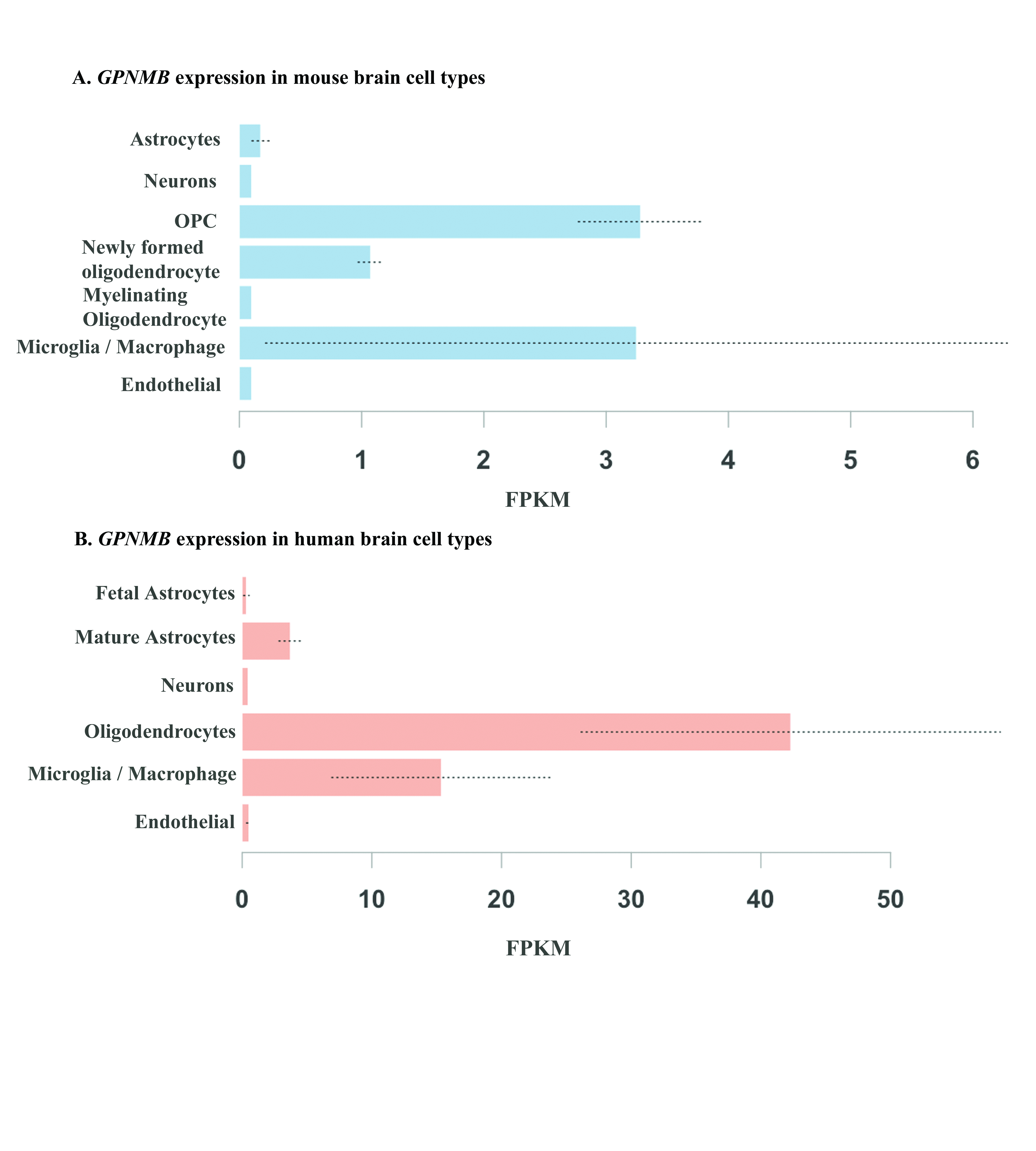

Supplement: Supplementary file 2 — High Resolution Image (TIFF 1104 kb) [file 10048_2017_514_MOESM1_ESM.tif]
